# Electrospun Conductive Nanofiber Yarn for a Wearable Yarn Supercapacitor with High Volumetric Energy Density

**DOI:** 10.3390/ma12020273

**Published:** 2019-01-16

**Authors:** Xianqiang Sun, Jianxin He, Rong Qiang, Nan Nan, Xiaolu You, Yuman Zhou, Weili Shao, Fan Liu, Rangtong Liu

**Affiliations:** 1 Henan Provincial Key Laboratory of Functional Textile Materials, Zhongyuan University of Technology, Zhengzhou 450007, China; liangjian791165@163.com (X.S.); qiangrong2009@126.com (R.Q.); 18037510635@163.com (N.N.); xiaoluyou0180@163.com (X.Y.); zhym_work@163.com (Y.Z.); liufan_a@hotmail.com (F.L.); ranton@126.com (R.L.); 2 Collaborative Innovation Center of Textile and Garment Industry, Zhengzhou 450007, China

**Keywords:** electrospinning, solid electrolyte, supercapacitor, PEDOT: PSS

## Abstract

One-dimensional, flexible yarn-shaped supercapacitors for woven cloth have the potential for use in different kinds of wearable devices. Nevertheless, the challenge that supercapacitors face is low energy density. In this paper, we present a low-cost and large-scale manufacturing method to construct a supercapacitor yarn with high power and high energy density. To construct the novel and flexible poly(3,4-ethylenedioxythiophene): poly(styrenesulfonate)–polyacrylonitrile (PDEOT: PSS-PAN)/Ni cotton (PNF/NiC) capacitor yarn, an electrospinning technique was initially used to wrap the polyacrylonitrile (PAN) nanofibers around the core Ni-coated yarn. The PEDOT: PSS–PAN nanofiber composite electrode was created using in situ deposition and H_3_PO_4_/PVA was used as a gel electrolyte. This electrode material has a yarn/nanofiber/PEDOT: PSS nanoparticle hierarchical structure, providing a high specific area and enhanced pseudocapacitance. The electrode demonstrated a high volumetric capacitance of 26.88 F·cm^−3^ (at 0.08 A·cm^−3^), an energy density of 9.56 mWh·cm^−3^, and a power density of 830 mW·cm^−3^. In addition, the PNF/NiC capacitor yarns are lightweight, highly flexible, resistant to bending fatigue, can be connected in series or parallel, and may be suitable for a variety of wearable electronic products.

## 1. Introduction

In recent years, wearable electronic devices have received significant attention for their potential in microelectronics, microrobots, electronic textiles, health monitoring, and medical devices, resulting in significant progress. However, a common problem of these wearable devices is how to develop a lightweight, high-performing, wearable energy-storage system. In energy storage devices, electrochemical capacitors have great development potential due to their high specific capacity, stable cycling performance, and good safety [1,2,3,4,5,6,7,8,9]. Early conventional supercapacitors that are bulky, rigid, and thick do not meet the wearable device requirements. In order to solve the problem of lightweight supercapacitors, electroactive materials, such as carbon fibers, carbon nanotubes, graphene, metal oxides, and conductive polymers, are widely used in the construction of flexible supercapacitors [10,11,12,13,14,15,16,17,18]. Wu et al. reported a paper-based supercapacitor based on electrochemical deposition, and the polyaniline nanofibers in the phase-separated composite material had an interconnecting net structure that contributed to a higher areal capacitance (8773 mF·cm^−2^ at 305.7 mA·cm^−2^) [19]. Wang et al. constructed a paper-based capacitor of *Cladophora* nanocellulose modified by glycidyltrimethylammonium chloride (EPTMAC). The modified nanocellulose has a dense and rough structure compared to conventional nanocellulose, resulting in an energy density as high as 3.1 mWh·cm^−3^ and a power density of 3 W·cm^−3^ [20]. Hao et al. used liquid-phase stripping to obtain single-layer and multi-layer black phosphor nanoparticles for assembly into paper-based capacitors. These nanoparticles were transparent and uniform in thickness, and their size was 10 to 100 nm for high volumetric capacitance (13.75 F·cm^−3^ at 0.01 V·s^−1^) and mechanical flexibility (15.5% capacity loss at 10,000 repeated bends) [21]. Obviously, two-dimensional paper-based flexible capacitors have the advantages of simple processing, good mechanical flexibility, and large-scale manufacturability.

One-dimensional linear supercapacitors have a greater development potential than two-dimensional paper-based supercapacitors due to the advantage of a large specific surface area and the ability to be woven into various structural fabrics [22,23,24,25,26,27,28,29]. Although current research has studied and prepared some high-performance, flexible one-dimensional linear supercapacitors, these supercapacitors are still very difficult to fabricate at low cost with the properties of being lightweight and flexible, having the ability to be woven into fabric, and possessing high power density and high energy density. Huang et al. used a stainless-steel wire to deposit a polyaniline material for high electrical conductivity, but the material had relatively low flexibility [30]. To solve this problem, Zhang et al. obtained a super-elastic electrochemical capacitor by winding a layer of carbon nanotubes on an elastic polyester filament and depositing an active material on top [31]. The capacitor has a specific capacity up to 111.6 F·g^−1^ at a 400% elongation and a current density of 0.5 A·g^−1^. The super-elastic capacitor is expensive and difficult to manufacture and cannot meet demands for large-scale production. Therefore, it is still a challenge to produce a low-cost, lightweight, high-capacitance electrochemical capacitor on a large scale. Chen et al. reported an electrochromic capacitor produced by depositing polyaniline in yarn that was wrapped in carbon nanotubes [32]. This capacitor electrode utilizes the changing color property of polyaniline; different colors (blue, green, and yellow) are displayed according to the voltage, and when it is charged and discharged. However, the fabrication cost increased because of the use of chemical vapor deposition for preparing the carbon nanotubes, which limited its large-scale industrial application.

Electrospinning, considered as a simple, low-cost process, is superior to other processes in synthesizing nanofibers with diameters ranging from tens of nanometers to several micrometers [33]. Electrospinning and subsequent carbonization processes have been used to prepare metal-oxide fibers such as TiO_2_, V_2_O_5_, and MnO_2_ [34,35,36]. Ahn et al. prepared TiO_2_ nanofiber thin films by electrospinning, and deposited RuO_2_ onto them by electrochemical deposition. The obtained composite electrode showed good rate performance [37]. Lee et al. obtained a RuO_2_-AgO_2_ nanofiber thin film electrode by electrospinning, and found that when the molar ratio of Ag and Ru precursor was 0.2, it had higher specific capacitance and good cycling performance (97%, at 300 cycles) [38]. Zhang et al. reported a Co_3_O_4_-CNFs thin film electrode by electrospinning. These interlaced nanofibers have porous structure and the corresponding composite electrode has higher specific capacitance (524 F·g^−1^, at 2 A·g^−1^) [39]. However, there has been little research on the preparation of electrospun flexible nanofiber core-spun yarn capacitor electrodes at present. Herein, we introduce a mass-production method for a flexible all-solid-state yarn-type supercapacitor by using Ni-coated cotton yarn as the core, wrapping the core with polyacrylonitrile (PAN) nanofibers prepared by electrospinning, depositing poly(3,4-ethylenedioxythiophene): poly(styrenesulfonate) (PEDOT: PSS) in situ, and using PVA/H_3_PO_4_ as a gel electrolyte. The nanofiber-wrapping structure has a large specific surface area and can provide more channels for electrolyte penetration and ion transfer, increasing the pseudocapacitance capacity of the composite electrode material. The capacitor, which is prepared by the hierarchical active material/nanofiber/yarn structure, has a high specific capacitance, good charge and discharge cycle stability, good bending performance, high fatigue resistance, high power density, high energy density, and can be woven into fabrics. These fabrics are expected to be applied to the next generation of wearable, functional, and intelligent electronic device components.

## 2. Materials and Experimental Methods

### 2.1. Materials 

All chemical reagents used in this work were of analytical grade (AR) and used without further purification. Polyacrylonitrile (PAN, Mw = 90,000) were purchased from J&K Scientific, Ltd. (Beijing, China) 3,4-ethylenedioxythiophene, sodium borohydride, and polystyrene sulfonate (PSS, Mw = 80,000) were purchased from Shanghai Aladdin Biochemical Technology Co., Ltd. (Shanghai, China). Sodium hydroxide, ammonium chloride, nickel sulfate hexahydrate, sodium citrate, sodium hypophosphite, ferric chloride, ethanol, N,N-dimethylformamide, and trichloromethane were purchased from Sinopharm Chemical Reagent Co., Ltd. (Shanghai, China). 

### 2.2. Preparation of Ni-Coated Cotton Yarns

The commercial cotton yarns were immersed in 0.25 mol·L^−1^ of a NaOH aqueous solution at 80 °C for 1 h and dried at 50 °C for 10 min to remove excess impurities. These precleaned cotton yarns were dipped into 50 mL of a 0.20 mol·L^−1^ nickel sulfate hexahydrate and 0.55 mol·L^−1^ hydrochloric acid mixed aqueous solution for 10 min, followed by dipping into 50 mL of a 0.26 mol·L^−1^ sodium borohydride and 0.25 mol·L^−1^ sodium hydroxide mixed aqueous solution for 10 min. The yarn was then washed with deionized water and placed in 100 mL of an electroless solution. The electroless solution was composed of nickel sulfate hexahydrate (NiSO_4_·6H_2_O, 0.12 mol·L^−1^), sodium hypophosphite (NaHPO_2_, 0.14 mol·L^−1^), ammonium chloride (NH_4_Cl, 0.84 mol·L^−1^), trisodium citrate (Na_3_C_6_H_5_O_7_, 0.10 mol·L^−1^), and ammonia (NH_3_·H_2_O, 2.5 mL).

### 2.3. Preparation of the Electrospun PAN Nanofiber Core-Spun Yarn

Nickel-coated yarns were used as the core yarns. Under a high-voltage environment, PAN nanofibers were wound on the Ni-coated yarns, resulting in a PAN core-spun yarn. The spinning voltage, distance, and speed were 15 kV, 16 cm, and 0.01 mm·min^−1^, respectively.

### 2.4. Fabrication of the PNF/NiC Composite Electrode

The PAN core-spun yarn was dipped in a chloroform solution of 3,4-ethylenedioxythiophene (50 mmol·L^−1^) for 30 min. Then, 5 mL of a 75 mmol·L^−1^ ethanol solution of ferric chloride was dripped onto the yarn using a dropper. The reaction was carried out at 20 to 25 °C for 6 h, 12 h, 24 h, 48 h, and 72 h. The yarn was then placed in a 50 mmol·L^−1^ polystyrene sulfonate aqueous solution for 20 min. After drying, PNF/NiC-6, PNF/NiC-12, PNF/NiC-24, PNF/NiC-48, and PNF/NiC-72 compound materials were obtained.

### 2.5. Assembly of the Yarn-Type Supercapacitor

All solid-yarn supercapacitors were assembled using the PNF/NiC composite yarn as the two electrodes and phosphoric acid (H_3_PO_4_)/polyvinyl alcohol (PVA) colloids as the gel electrolyte. The H_3_PO_4_/PVA gel electrolyte was synthesized by mixing 6 g PVA and 9 g H_3_PO_4_ in 50 mL deionized water at 80 °C for 2 h under vigorous stirring. The PNF/NiC composite electrodes were then coated with the H_3_PO_4_/PVA gel electrolyte and dried at room temperature for 24 h. The two compound yarns were twisted together to form a yarn-type supercapacitor.

### 2.6. Material Characterization

The surface morphologies and structure of the sample were characterized by scanning electron microscopy (SIGMA 300, Zeiss Merlin Compact field emission scanning electron microscopy, Jena, Germany) and transmission electron microscopy (JEM-2011 transmission electron microscope, JEOL, Akishima, Tokyo). The nitrogen adsorption-desorption experiment (Autosorb-iQ automatic specific surface and aperture distribution analyzer, Quantachrome, Boynton Beach, FL, USA) was used to record the specific surface area of the material. The X-ray photoelectron spectroscopy tests (monochromated X-ray photoelectron spectrometer, XPS, Model 558, PerkinElmer, Waltham, MA, USA) of composites was carried out. Fiber diameter histogram statistics were from field emission scanning electron microscope (Phenom LE, Eindhoven, The Netherlands).

### 2.7. Electrochemical Measurement

All electrochemical tests, including the galvanostatic charge and discharge measurements, cyclic voltammograms, cycling performance, and electrochemical impedance spectroscopy was tested by a CHI 660A electrochemical workstation (CH instrument, Austin, TX, USA). Electrochemical performance parameters of the capacitor were measured by a two-electrode system.

## 3. Results and Discussion

### 3.1. Structure and Morphology Characterization

**Figure 1:**
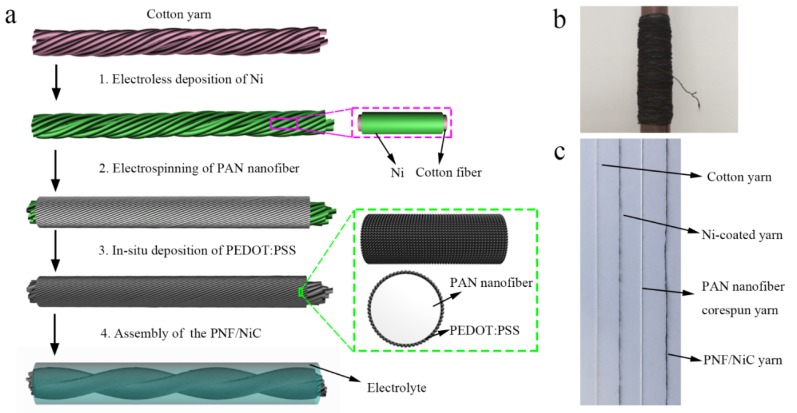
(**a**) Schematic illustration of the fabrication of the PNF/NiC capacitor yarn. (**b**) Photograph of the 500 m long Ni-coated cotton yarn wound on the spinning cone. (**c**) Photograph of various yarns during processing.

The fabrication process of the PNF/NiC capacitor yarn is shown in Figure 1a. Commercial cotton yarns were coated with a nickel layer by chemical polymerization to form conductive yarns, which can act as current collectors, and also maintain the flexibility of the textiles. In this study, a large number of high surface area PAN nanofibers prepared by electrospinning were wrapped on the core Ni-coated yarn to form PAN-nanofiber core-spun yarn (Figure S1, Supplementary Materials). PEDOT: PSS nanoparticles were deposited on PAN nanofibers by in situ polymerization, through which a PNF/NiC compound yarn was formed. The PNF/NiC yarns, which were used as the positive and negative electrodes, were soaked with the PVA/H_3_PO_4_ solution and allowed to solidify at room temperature. The resulting positive and negative electrode materials were twisted together to form a PNF/NiC capacitor yarn. This method can produce a large number of PAN nanofibers with a large specific surface area, and it can provide more channels for electrolyte penetration and ion transfer, thus improving the capacitance of the composite material. Figure 1b shows a photograph of a 500-m long Ni-coated cotton yarn that was wound on a spinning cone. Figure 1c is a photograph of cotton yarn, Ni-coated yarn, PAN nanofiber core-spun yarn and PNF/NiC yarn.

**Figure 2:**
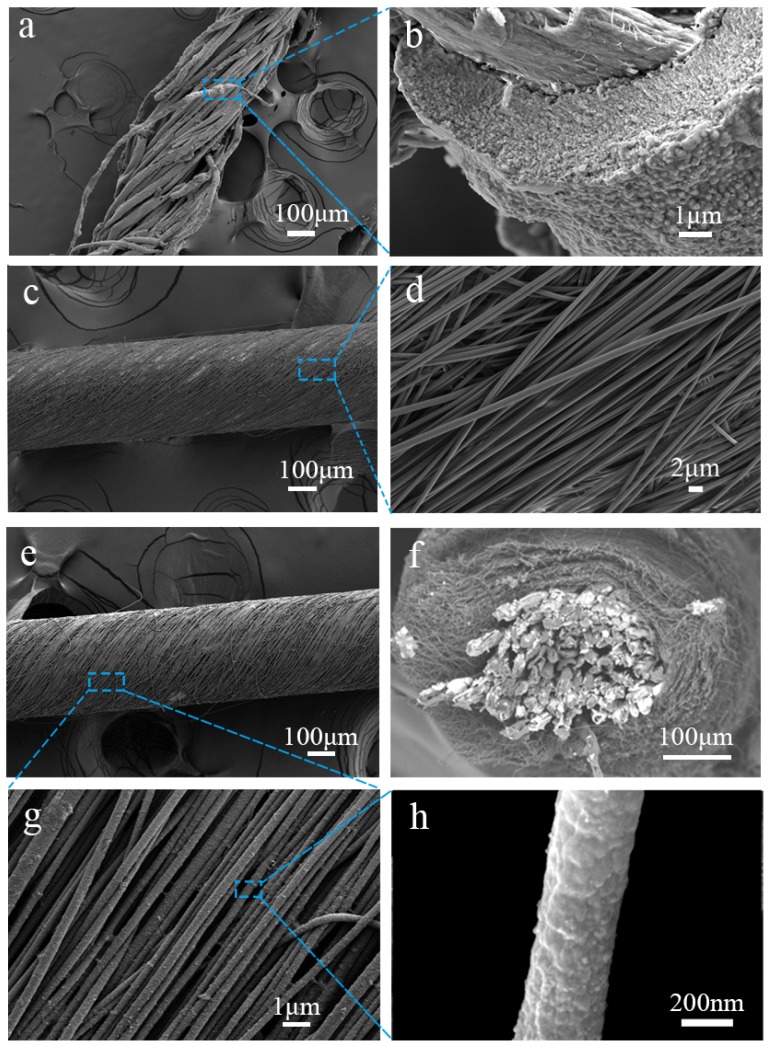
(**a**) Ni-coated yarn, and (**b**) cross-section of a single fiber. (**c**) Polyacrylonitrile (PAN) nanofiber core-spun yarn at (**d**) high magnification, and (**f**) its cross-section. (**e**) PNF/NiC -24 electrode yarn at (**g**) high magnification, and (**h**) a single nanofiber.

In order to determine the microstructure of the material, we characterized it by scanning electron microscope (SEM). The SEM images in Figure 2 show that the nickel layer was uniformly and densely coated on the surfaces of the cotton fibers; the average diameter of the nickel-coated cotton yarn was 250 μm (Figure 2a), and the thickness of the nickel layer was approximately 2.3 μm (Figure 2b). The density of the Ni-coated yarn was much lower than pure wire or metal yarns previously reported in the literature [19]. For instance, the density of pure metal wire is 21.45 g·cm^−3^ (platinum wire), and electroless of Ni only weighs the cotton yarns with 344%, the density of the Ni-coated cotton yarn was 4.00 g·cm^−3^ (the density of the cotton yarn was 0.90 g·cm^−3^, which is only 22.5% of the Ni-coated yarn). Most importantly, the Ni-coated yarn had extremely high electrical conductivity (1.10 Ω·cm^−1^) while maintaining the softness of the textiles (Figure S2a, Supplementary Materials). The resistance increased linearly along the length of Ni-coated yarn, indicating that the nickel layer was uniform along the length (Figure S2b, Supplementary Materials). This is important for the preparation of long, continuous, and linear capacitor yarns, which can ensure a continuous transfer of charge. 

**Figure 3:**
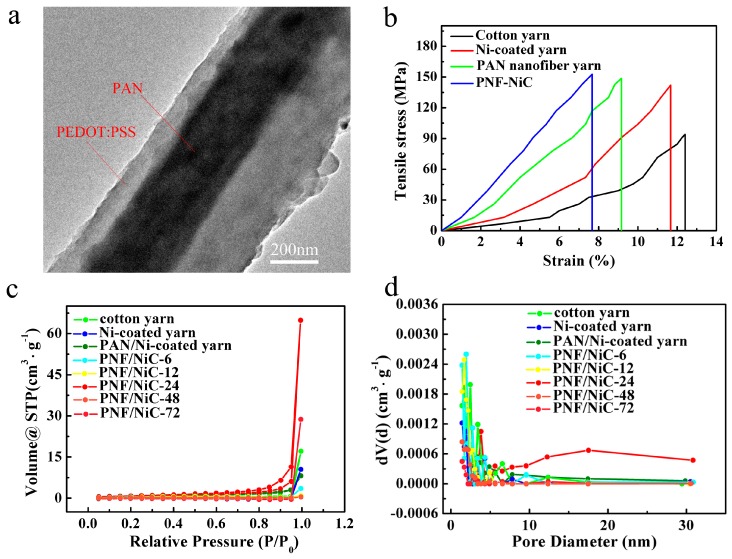
(**a**) TEM image of PNF/NiC-24. (**b**) Comparison of stress-strain curves of cotton yarn, nickel-coated cotton yarn, PAN nanofiber core-spun yarn, and PNF/NiC yarn. (**c**) Nitrogen adsorption isotherms and (**d**) Barrett-Joyner-Halenda pore size distribution curves of cotton and nickel-coated yarn, PAN nanofibers, and PNF/NiC compound cotton yarn at different processing times.

In order to prepare the PEDOT: PSS-coated and nanofiber-wrapped Ni-coated yarn composite electrode (PNF-NiC) PAN nanofibers were first wrapped over the Ni-coated yarn surface by conjugate electrospinning (Figure 2c). The PAN nanofibers were parallel and spirally distributed on the Ni-coated yarn, as shown in Figure 2c,d. Nanofibers with a size of 389 nm were densely wrapped in parallel around the yarn surface in a specific twist direction, and the cotton yarn was completely wrapped inside. The nitrogen adsorption and desorption test of Ni-coated yarn showed that the specific surface area of the PAN nanofiber wrapped Ni-coated yarn increased steadily (from 1.755 to 3.549 m^2^·g^−1^). Obviously, this is conducive to improving the load of the electrochemical active PEDOT: PSS material. Thus, these nanoscale fibers have a large specific surface area, can be used as templates, and could be applied to in situ polymerization of PEDOT: PSS. A PEDOT: PSS nanofiber yarn with a weight gain of 0.33 mg·cm^−1^ was produced. After in situ polymerization of PEDOT: PSS, the surface of the nanofiber-wrapped yarn did not change significantly as illustrated by the low magnification SEM images, and the nanofiber maintained a stable, parallel twist structure. The nanofibers were parallel to each other, without disruption or crossover (Figure 2e,g). As shown in the high magnification SEM image, at a deposition time of no more than 24 h, PEDOT: PSS was uniformly deposited on the surface of the PAN nanofiber template. The surface morphology of PEDOT: PSS with a deposition of less than 24 h was a nanoparticle-wrapped dense structure. When deposited for 48 h, a rough morphology was present due to excessive deposition; the nanofibers were wrapped with PEDOT: PSS nanoparticles of uneven particle sizes. By further increasing the deposition time to 72 h, a dendritic structure was generated and the nanofiber coating was very uneven (Figure S3d, Supplementary Materials). The average diameters of PAN nanofibers ranged from 460 to 730 nm at the deposition time of 6 to 72 h. The thickness of single PAN nanofibers with in situ deposition of PEDOT: PSS increased with increasing deposition time (Figure S4, Supplementary Materials). In addition, the average coating thickness of PEDOT: PSS was 190 nm after 24 h of deposition (Figure 3a).

The presence of PEDOT: PSS in the PAN nanofiber composite yarns was confirmed by X-ray photoelectron spectroscopy (XPS). After in situ deposition of PEDOT: PSS, the C, N, O, S, and Na elements were clearly observed in the broad sweep spectrum (Figure S5a, Supplementary Materials). Meanwhile, The XPS spectrum of S 2p shows that the electron binding energy was in the band of 166 to 170 eV assigned to the 2p orbital of the sulfur atoms from the sulfonic acid group in PSS. The two XPS bands with electron binding energies between 162 and 166 eV belonged to the 2p orbital of the sulfur atoms from the thiophene group in PEDOT [29].

Compared with PAN nanofibers, the specific surface area and pore volume increased with the increase of the in situ deposition time of PEDOT: PSS onto the PAN nanofibers. The nitrogen adsorption–desorption curve was V-shaped, indicating that the PEDOT: PSS-polymerized nanofibers contained open pores, which can have a capillary absorption effect (Figure 3). When the in situ deposition time increased, the specific surface area and pore volume of the obtained PNF/NiC electrode significantly increased. The pore structure parameters of the in situ deposited PEDOT: PSS electrode material at different times are shown in Supplementary Table S1. However, when the in situ deposition time exceeded 24 h, both the specific surface area and pore volume peaks appeared and then decreased. The PNF/NiC nanofiber composite electrodes with 24 h in situ deposition were mainly composed of 3 nm and 17 nm mesopores, with a total pore volume and specific surface area of 0.018 cm^3^·g^−1^ and 5.329 m^2^·g^−1^, resepectively. The composite had a large specific surface area in the mesoporous structure, could enhance the electrochemical reaction sites, and made the electroactive material have a sufficiently large specific surface area to participate in the charge-transfer reaction. Thus, the composites enhanced the electrochemical capacitance.

As shown in the stress-strain curves, the tensile strength of the Ni-coated yarn (141.96 MPa) was greater than the initial cotton yarn (94.12 MPa) (Figure 3b). To evaluate the resistance fatigue of the Ni-coated yarn under wearable conditions, the yarn was repeatedly bent and released 3000 times under a small bending radius (r = 1 mm), resulting in only a 5% increase in resistance (Figure S6, Supplementary Materials). In contrast, the composites still showed excellent mechanical toughness and could withstand 500 repeated bends. The resistance increased by only 28% with a smaller bending diameter (r = 1 mm) (Figure S7, Supplementary Materials). The PNF/NiC composite electrode prepared in this study had better tensile properties than the Ni-coated yarn, and stress and strain tests showed that the tensile strength of PNF/NiC composite electrode (152.75 MPa) was 161% greater than the Ni-coated yarn and approximately twice as high as pure cotton yarn.

The higher conductivity of the electrode material helped reduce charge loss during transfer. The ratio of EDOT to PSS and the deposition time significantly influenced the conductivity of the material itself; at a molar ratio of EDOT to PSS of 1:1.5, the PNF/NiC electrode had the highest conductivity (Figure S8, Supplementary Materials). In addition, when the deposition time exceeded 24 h, the conductivity maintained a steady increase, then a slight decrease (Figure S9, Supplementary Materials). 

### 3.2. Electrochemical Performance

**Figure 4:**
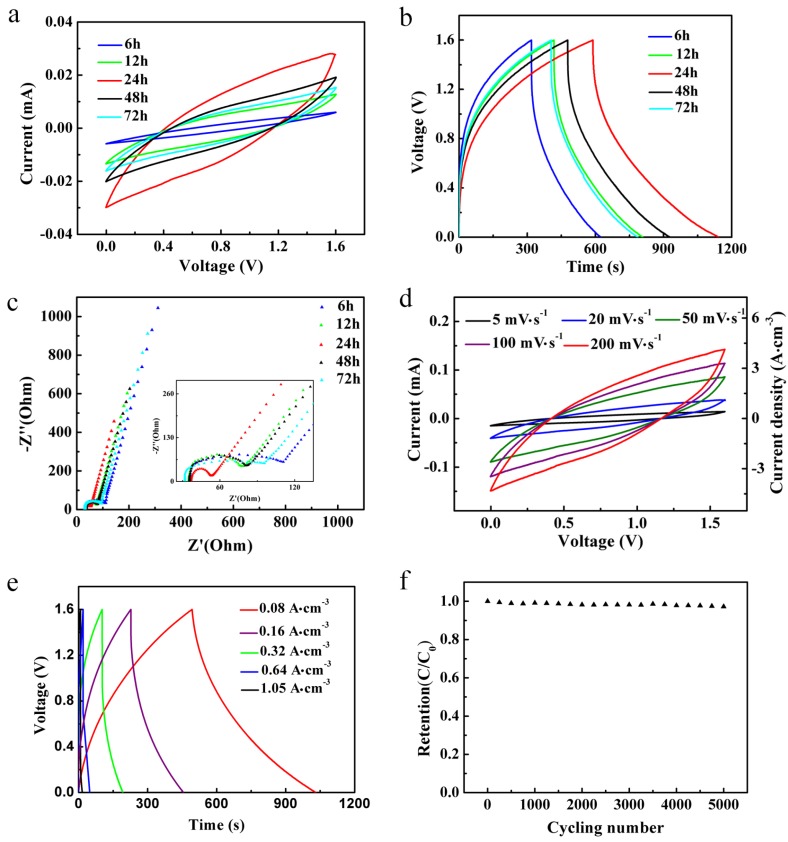
(**a**) Cyclic voltammetry curves, (**b**) galvanostatic charge and discharge curves, and (**c**) Nyquist impedance diagrams (frequency from 0.01 Hz to 100 kHz, Voltage DC = 0 vs. open circuit) of the PNF/NiC-6, PNF/NiC-12, PNF/NiC-24, PNF/NiC-48, and PNF/NiC-72 electrodes. (**d**) Cyclic voltammetric curves, (**e**) cell voltage vs time under galvanostatic charge–discharge, and (**f**) capacity retention (at 5000 charges and discharges, at 1.05 A·cm^−3^) of the PNF/NiC-24 SC yarn.

In order to evaluate the electrochemical performance of the PNF/NiC composite electrode, PVA/H_3_PO_4_ gel was used as an electrolyte and separator, and a pair of PNF/NiC composite electrode materials was assembled for a wearable supercapacitor yarn. Figure 4a shows the cyclic voltammetry curves of a PNF/NiC composite yarn capacitor that was deposited in situ with PEDOT: PSS at different times at a scanning rate of 20 mV·s^−1^ using a two-electrode system. Except for the PEDOT: PSS sample from an in situ deposition of 6 h, the PNF/NiC yarn composite capacitors all showed slightly tilted rectangular-like shapes. The PNF/NiC-24 capacitor (SC) had the largest area, corresponding to higher specific capacitance. Because the mass of the electroactive material absorbed on the electrode material was negligible compared to the weight of the entire device, the volumetric capacitance of yarn-type capacitors could better describe the performance of the supercapacitor compared to the gravimetric capacitance. In the actual calculation, we considered the composite yarn electrode as a cylinder, and the diameter of the cross-section as the thickness of the composite electrode yarn. Herein, the SC yarn, which was 5 cm long with two composite electrode yarns and a surrounding solid electrolyte, was 0.005 cm^3^. The volumetric capacitance of the PNF/NiC composite electrode yarn was calculated according to the following equations:

(1) $$C_{v,capacitor}=\frac{I\times \Delta t}{2\times V_{\;yarn}\times \Delta U}\;\;,$$

(2) $$\frac{1}{C_{v,capacitor}}=\frac{1}{C_{v,electrode}}+\frac{1}{C_{v,electrode}}.$$

$C_{v,capacitor}$ is the volumetric capacitance of the capacitor device in the two-electrode system, *I* refers to the discharge current, ∆*t* refers to the discharge time, $V_{\mathrm{yarn}}$ refers to the effective active material volume of the single PNF/NiC composite electrode yarn, ∆U refers to the voltage window, and $C_{v,electrode}$ is the volumetric capacitance of a single PNF/NiC composite yarn. Figure 4b shows the galvanostatic charge-discharge curves of PNF/NiC SC at a current density of 0.08 A·cm^−3^; the capacitors with PEDOT: PSS were deposited at different times, and all exhibited a triangular shape. The volumetric capacitance values of PEDOT: PSS deposited at different times, which were calculated from the galvanostatic charge and discharge curves, are summarized in Supplementary Table S2. PNF/NiC yarn electrode with PEDOT: PSS deposited for 24 h in situ had the highest specific capacitance of 55.36 F·cm^−3^. This was consistent with the cyclic voltammetry data; further increasing the time for in situ deposition led to a decrease in the specific capacitance of the capacitor. This is due to the excessive deposition of PEDOT: PSS to form a dendritic [19,20] structure, which is formed by the aggregation of PEDOT: PSS nanoparticles.

Figure 4c and the illustration of the Nyquist plot in a magnified high-frequency region illustrate small semicircles, which were mainly due to the small charge transfer resistance and a Weber curve in the low-frequency region, indicating the ideal capacitive behavior of the device. PNF/NiC capacitors with in situ deposition of PEDOT: PSS for 24 h exhibited a significantly lower 18 Ω-resistance and the shortest Weber area compared to other samples, indicating the best ion diffusion rate due to smaller contact resistance between the PNF/NiC yarns and electrolyte ions. The resistances of PNF/NiC capacitors with different in situ deposition times are shown in Supplementary Table S3. The good electrochemical activity and low resistance of PNF/NiC deposited for 24 h was mainly attributed to the deposition amount of PEDOT: PSS nanoparticles reaching a high level, resulting in a relatively high conductivity at maximum specific surface area. However, when the in situ deposition time exceeded 24 h, the amount of PEDOT: PSS deposited was too large, hindering the diffusion of the electrolyte and resulting in low capacitance and high resistance. Therefore, the in situ deposition of PEDOT: PSS for 24 h showed a more ideal capacitive performance.

As expected, the cyclic voltammetry curves of the PNF/NiC-24 electrode yarn at a scanning rate of 5 to 200 mV·s^−1^ showed slightly sloped rectangular-like shapes. The cyclic voltammetry curve was not significantly distorted at high scanning rates, indicating that ions were efficiently transferred in the electrode material and a high rate capability (Figure 4d) [19,29]. The galvanostatic charge and discharge curves at different current densities all exhibited triangular shapes with a coulombic efficiency of 94%, indicating the nanofiber yarn electrode material had excellent reversibility and good charge transportation between the two electrodes. In addition, the PNF/NiC-24 SC electrode showed that the capacitor still maintained 97% of the initial capacitance after 5000 continuous charge and discharge cycles (Figure 4f).

There was an electrochemical effect on the pseudocapacitance due to underpotential deposition (UPD) of electroactive material on the electrode yarn with a highly reversible adsorption-desorption reaction. Therefore, the specific capacity was related to the amount of PEDOT: PSS on the surface of the nanofibers; with increased time, the amount of PEDOT: PSS deposited on the surface of the nanofibers increased, and the electrochemical performance was increased. The 24-h deposition-time sample exhibited the largest capacitance performance (Figure S10, Supplementary Materials). Despite increasing the deposition time, the capacitors exhibited attenuating electrochemical performance despite more PEDOT: PSS on the surface of the nanofibers. As the deposition time exceeded 24 h, the thickness of the deposited PEDOT: PSS increased, which increased the rough dendritic structure. The specific surface area of the electrode material also decreased significantly, increasing the discontinuity of the electron transmission.

**Figure 5:**
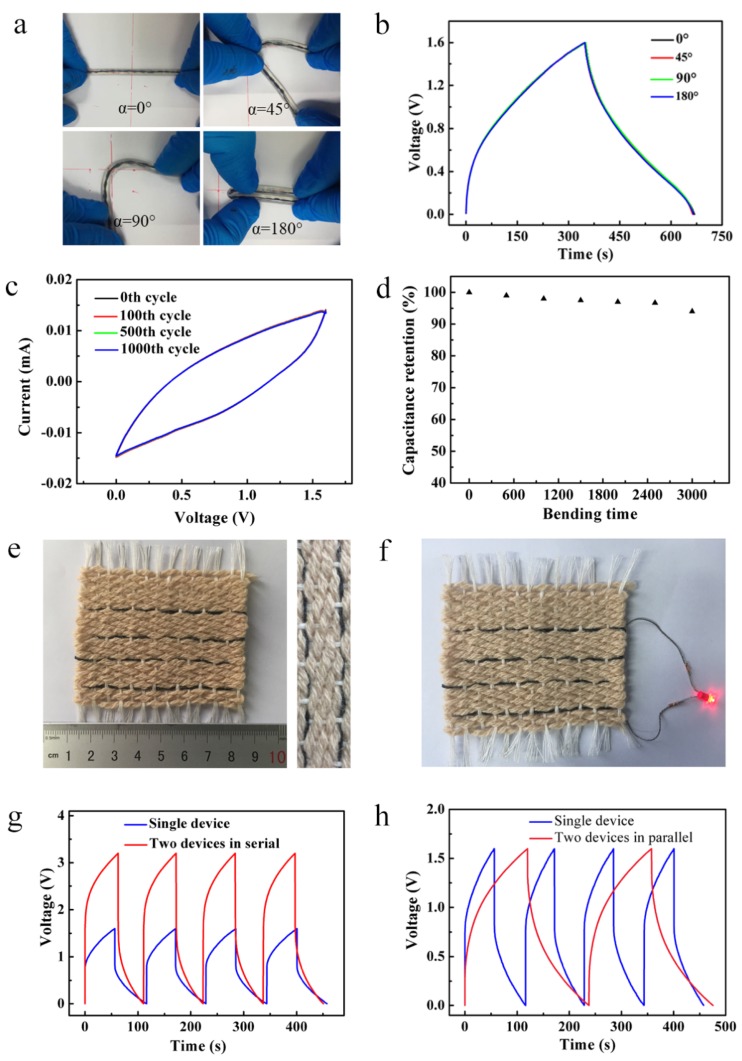
(**a**) Silicone tube used for easy bending of PNF/NiC SC yarn at different angles and (**b**) its corresponding galvanostatic charge and discharge curves. (**c**) Cyclic voltammetry curves of PNF/NiC SC yarns, which were bent for 100, 500, and 1000 cycles under a 180° bending angle. (**d**) Capacity retention of SC yarns at 3000 bending cycles. (**e**) PNF/NiC SC yarns woven into fabric and (**f**) lighting a diode. (**g**) Galvanostatic charge and discharge curves of PNF/NiC SC yarn in series and (**h**) parallel.

As mentioned before, our SC yarns, which are based on traditional cotton yarn processing, had good spinnability and mechanical properties, and the linear capacitors assembled with gel electrolytes were ideal yarns for wearable fabrics. Two woven capacitors composed of yarn and electrolyte were woven into fabric to verify the wearable stability. The PNF/NiC capacitor with in situ deposition of 24 h was used to test the bending properties at 0° (unbent) to 180° (folded), and the galvanostatic charge and discharge curves shown in Figure 5b indicate that the device that bends arbitrarily has little effect on the capacitive behavior. The SC yarn was bent for 3000 cycles to simulate a daily wearable condition. Over the entire bending test, the specific capacitance only decreased by 6%. Therefore, the wearable PNF/NiC capacitors had good fatigue durability, which was mainly attributed to the flexibility of the PNF/NiC electrode, and the penetration of the gel electrolyte played a role in protecting the original electrode.

The PNF/NiC capacitors can be made into fabric in series or parallel to meet the different wearable conditions. The capacitor with a single SC yarn operated at 1.6 V was compared to two and three SC yarns connected in series. The galvanostatic charge and discharge curves show that the charge­-discharge voltage window of the SC yarn increased by a factor of two and three respectively, the discharge time was unchanged, and it had good extensibility (Figure 5g and Figure S11a, Supplementary Materials). When in two and three parallel connections, the galvanostatic charge and discharge curves show that the output current of the capacitor was increased by two and three times, respectively, and the discharge time also increased two and three times (Figure 5h and Figure S11b, Supplementary Materials). The SC yarns can be connected in series and in parallel to meet the wearable operating voltage requirements and different operating capacitance requirements on different conditions. In addition, two SC yarns in series were able to light LEDs with a minimum operating voltage of 1.7 V (Figure 5f). Therefore, the SC yarns we constructed have the potential to be used in wearable device fabrics.

**Figure 6:**
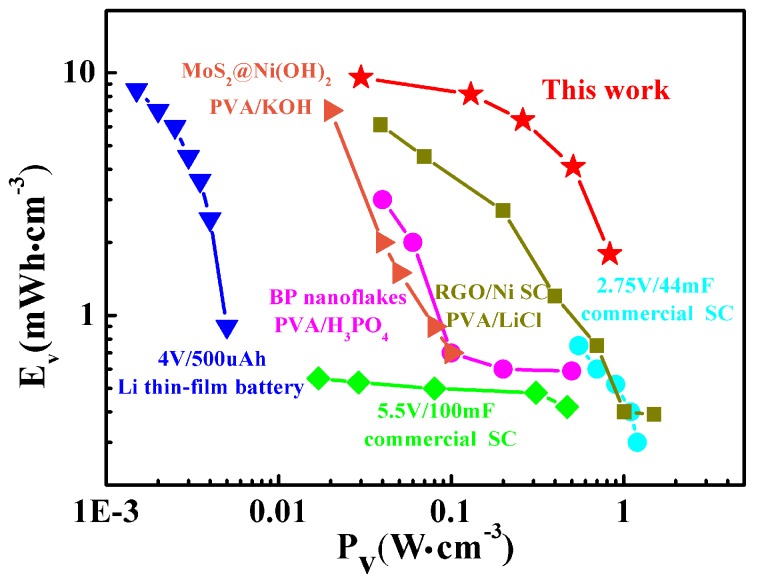
Ragone graph of different supercapacitor yarns and commercial examples.

The power and energy densities of an electrochemical capacitor are important indicators for materials in practical applications. Herein, the energy density and power density of the developed SC yarns, some commercial capacitors, and supercapacitors reported recently in the literature were used for comparison. As shown in Figure 6, the SC yarn we made is in the upper right corner of the graph, and the maximum energy density was 9.56 mWh·cm^−3^, which is approximately 12 times a commercial 2.75 V/44 mF [40] capacitor, 17 times a commercial 5.5 V/100 mF [41] capacitor, and equivalent to a 4 V/500 μAh thin-film lithium battery [42,43]. The maximum energy density of the SC yarn was two times that of the black phosphorus nanoparticle paper-based all-solid-state supercapacitor [21], one times that of the MoS_2_@Ni(OH)_2_ [44], an all-solid-state supercapacitor, and two times that of the RGO/Ni-coated yarn-shaped capacitor [16]. The maximum power density of our constructed SC yarn was 830 mW·cm^−3^. Considering the above evidence, the constructed SC yarn was greater than other published data in energy density.

## 4. Conclusions

In this article, we report a novel yarn-shaped supercapacitor with high energy density and high power density. We converted ordinary cotton yarns into conductive electrodes by an electroless method and wrapped these core yarns with PAN nanofibers by electrospinning. A flexible yarn with energy storage capability was obtained by in situ deposition of PEDOT: PSS and integration with a gel electrolyte. The electrochemical performance of SC yarns reached or exceeded previously reported solid or yarn-shaped capacitors. The yarn demonstrated high volumetric capacitance (26.88 F·cm^−3^, 25.31 F·g^−1^), excellent cycling stability (capacitance retention was 97% at 5000 cycles), high energy density (9.56 mWh·cm^−3^), high power density (830 mW·cm^−3^), as well as good flexibility and fatigue durability (capacitance retention was 94% at 3000 cycles of bending at 180°). In addition, the low-cost SC yarns can be connected in series and in parallel when woven into fabrics to meet different wearable requirements. The yarns are expected to be ideal energy storage devices, which can be applied to the next generation of flexible, woven electronic products. 

## Supplementary Materials

The following are available online at http://www.mdpi.com/1996-1944/12/2/273/s1, Figure S1: Electrospinning process diagram of nanofiber core yarn, PAN nanofiber core-spun yarn diagram; Figure S2: I-V curves of 5 cm long nickel-coated yarns with different nickel coating times, Variations of resistance of nickel-coated cotton yarns with lengths; Figure S3: Electron micrographs of single nanofibers of PNF/NiC-6, PNF/NiC-12, PNF/NiC-48, PNF/NiC-72; Figure S4: SEM graph and diameter distribution histogram of PAN, PNF/NiC-6, PNF/NiC-12, PNF/NiC-24, PNF/NiC-48, PNF/NiC-72 compound material; Figure S5: XPS spectrum of PNF/NiC composite cotton yarn, XPS spectrum of S 2p; Figure S6: Resistance increase of Ni-coated cotton yarn after 3000 bending cycles; Figure S7: Bending curves of 500 cycles of PNF/NiC-24 composite yarns; Figure S8: Conductivity curves of PNF/NiC-24 composite yarns with different mole ratios (mol:mol) of EDOT and PSS, Figure S9: Conductivity curves of different deposition time for PAN nanofiber core-spun yarns; Figure S10: Volumetric capacity curve after in-situ deposition of PEDOT:PSS for different hour; Figure S11: Galvanostatic charge and discharge curves of three SC yarns in series and parallel; Table S1: Nitrogen absorption and desorption data for different stage’s materials; Table S2: Specific capacitance of yarn electrode material deposited PEDOT:PSS for different hours; Table S3: Migration resistance of capacitors for different deposition time of PEDOT:PSS.
