# Supplementary material for: Electrospun Conductive Nanofiber Yarn for a Wearable Yarn Supercapacitor with High Volumetric Energy Density
Source: Materials (Basel). 2019 Jan 16;12(2):273. doi: 10.3390/ma12020273 (PMC6356552; doi:10.3390/ma12020273)
Supplement: Supplementary file 1 [file materials-12-00273-s001.pdf]

materials

Supplementary Information

Electrospun Conductive Nanofiber Yarn for a Wearable Yarn Supercapacitor with High Volumetric Energy Density

Xianqiang Sun [1,2], Jianxin He [1,2,*], Rong Qiang [1,2], Nan Nan [1,2], Xiaolu You [1,2], Yuman Zhou [1,2], Weili Shao [1,2,*], Fan Liu [1,2] and Rangtong Liu [1,2]

1 Henan Provincial Key Laboratory of Functional Textile Materials, Zhongyuan University of Technology, Zhengzhou 450007, China; liangjian791165@163.com (X.S.); qiangrong2009@126.com (R.Q.); 18037510635@163.com (N.N.); xiaoluyou0180@163.com (X.Y.); zhym_work@163.com (Y.Z.); liufan_a@hotmail.com (F.L.); ranton@126.com (R.L.)

2 Collaborative Innovation Center of Textile and Garment Industry, Zhengzhou 450007, China

* Correspondence: hejianxin771117@163.com (J.H.); weilishao@163.com (W.L.)

Electrochemical performance calculation

For all solid state SCs, the volumetric specific capacity (C_v) and mass specific capacity (C_m) calculations for the entire device follow the following equation:

$$C_V = \frac{I \times \Delta t}{V \times \Delta U}, \quad (1)$$

$$C_m = \frac{I \times \Delta t}{m \times \Delta U}, \quad (2)$$

Here, I, Δt, and ΔU represent the discharge current, discharge time, and potential window, respectively. V represents the volume of the two yarns and the surrounding electrolyte of the entire device, and m represents the mass of effective active material for the capacitor electrode material.

According to the constant current charge–discharge curves at different current densities, the volumetric energy density E_v and the power energy density P_v can be calculated, following the following equation:

$$E_V = \frac{1}{2} \times C_V \times \Delta U^2, \quad (1)$$

$$P_V = E_V / \Delta t. \quad (1)$$

Supplementary Figures

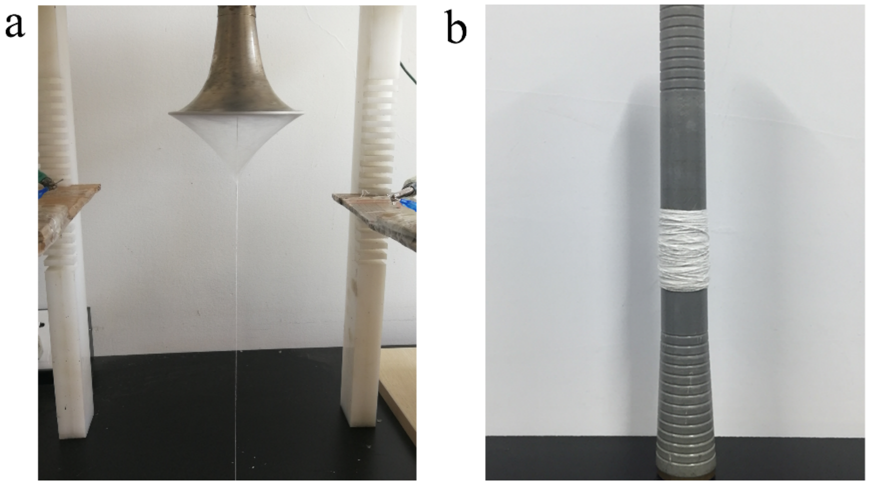

Figure S1. (**a**) Electrospinning process diagram of nanofiber core yarn, (**b**) PAN nanofiber core-spun yarn diagram.

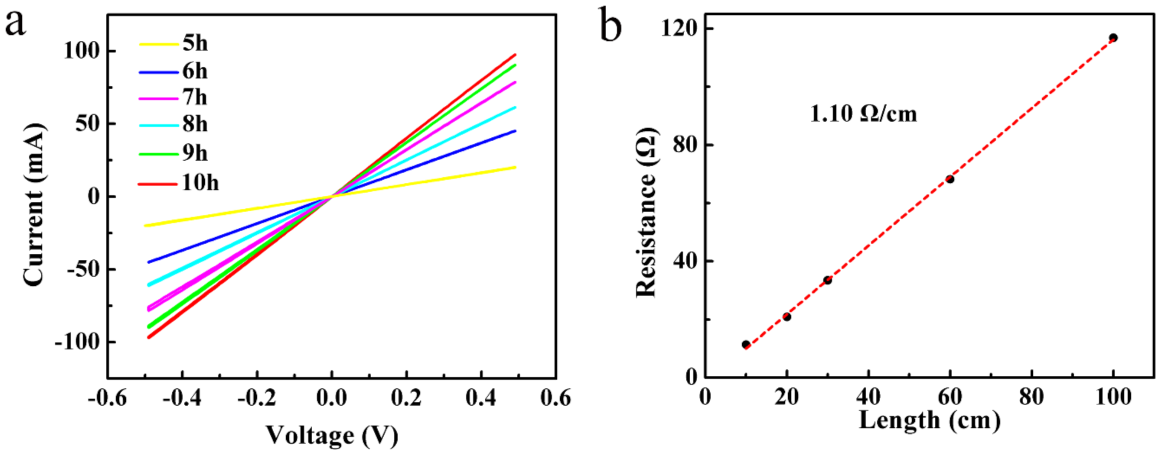

Figure S2. (a) I-V curves of 5 cm long nickel-coated yarns with different nickel coating times. (b) Variations of resistance of nickel-coated cotton yarns with lengths.

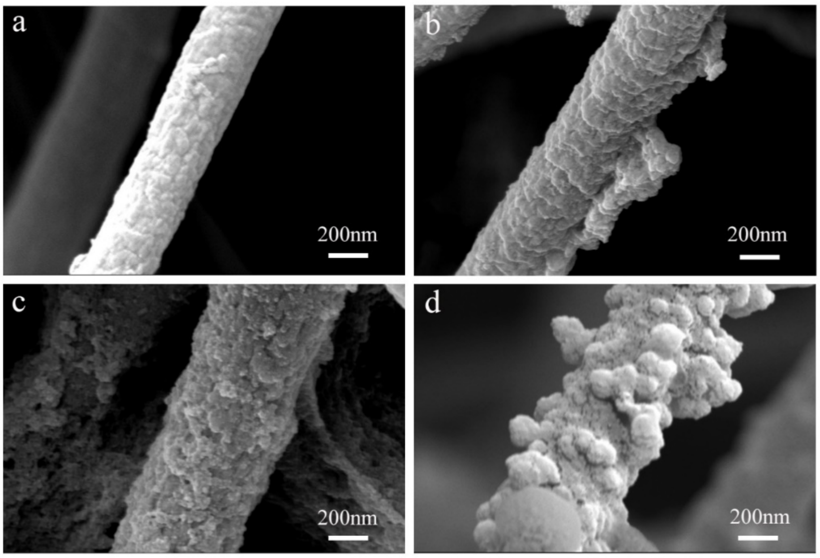

Figure S3. (**a**) Electron micrographs of single nanofibers of PNF/NiC-6, (**b**) PNF/NiC-12, (**c**) PNF/NiC-48, (**d**) PNF/NiC-72.

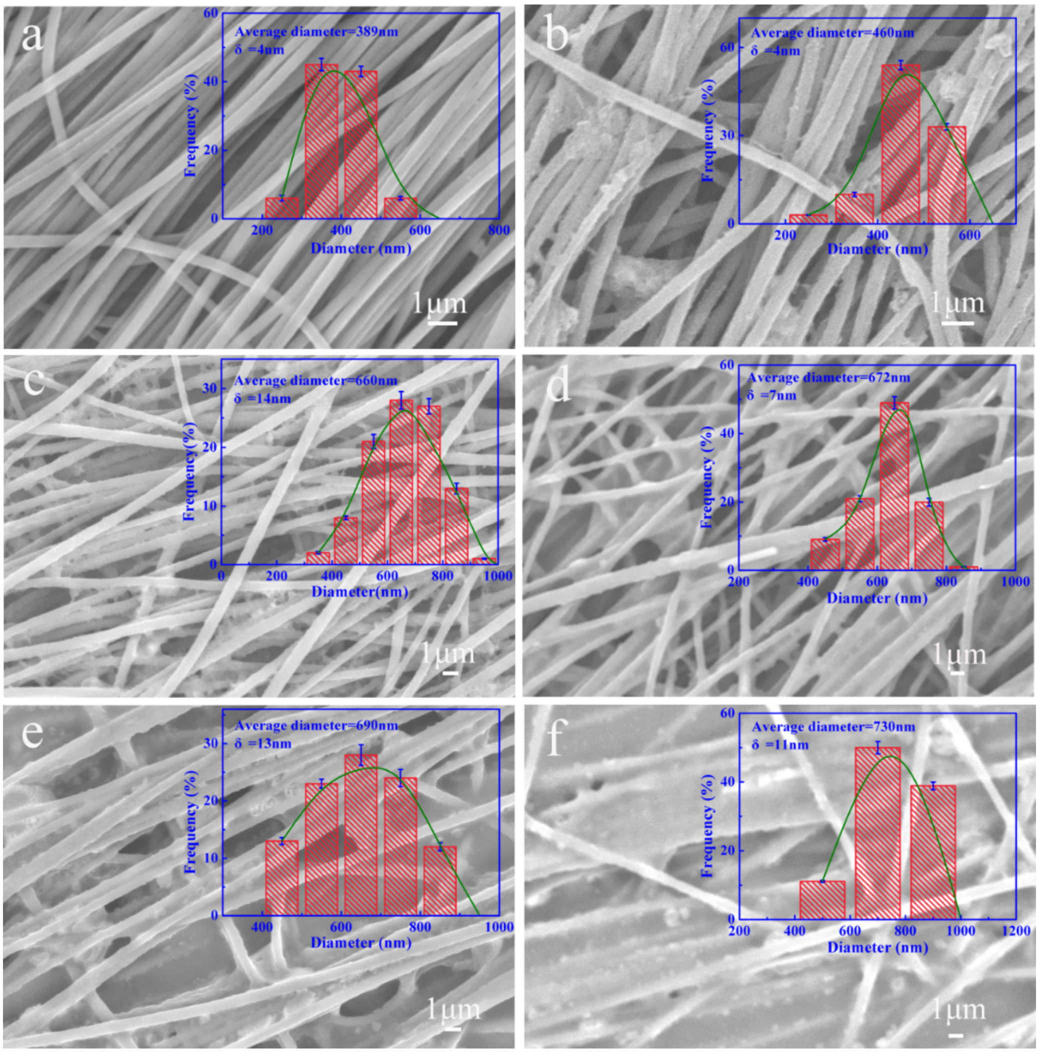

Figure S4. SEM graph and diameter distribution histogram of (**a**) PAN, (**b**) PNF/NiC-6, (**c**) PNF/NiC-12, (**d**) PNF/NiC-24, (**e**) PNF/NiC-48, (**f**) PNF/NiC-72 compound material.

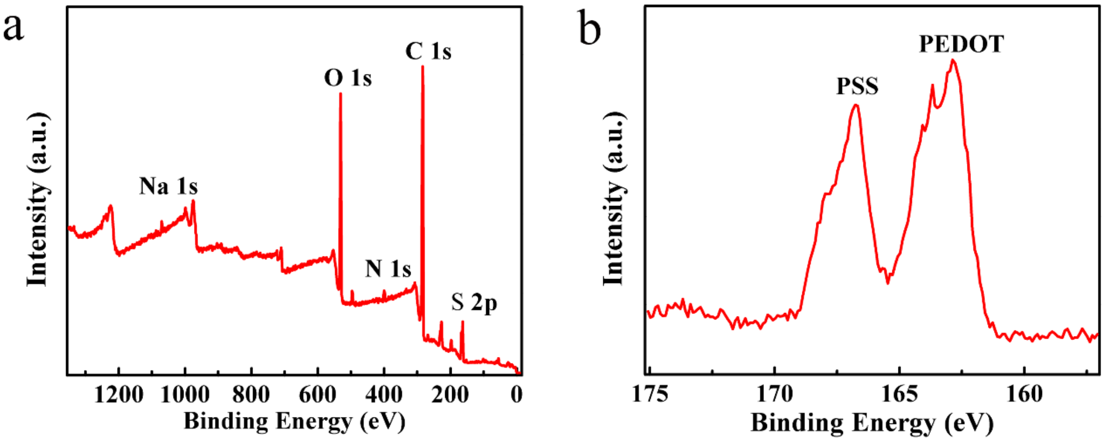

Figure S5. XPS spectrum of PNF/NiC composite cotton yarn (**a**), XPS spectrum of S 2p (**b**).

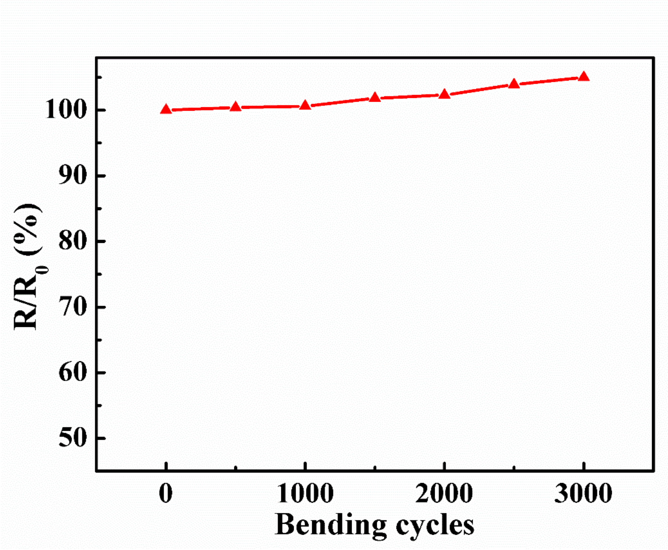

Figure S6. Resistance increase of Ni-coated cotton yarn after 3000 bending cycles.

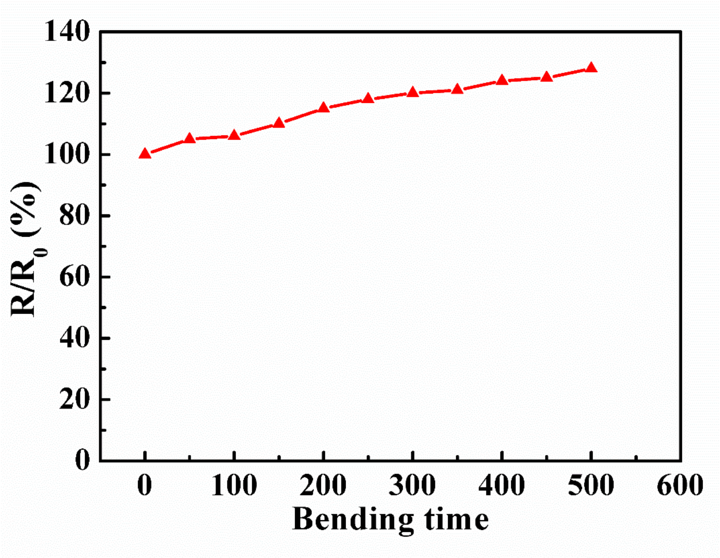

Figure S7. Bending curves of 500 cycles of PNF/NiC-24 composite yarns.

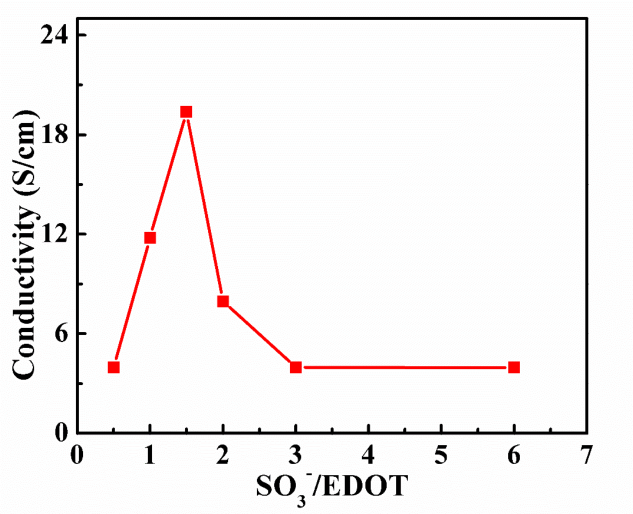

Figure S8. Conductivity curves of PNF/NiC-24 composite yarns with different mole ratios (mol:mol) of PEDOT and PSS.

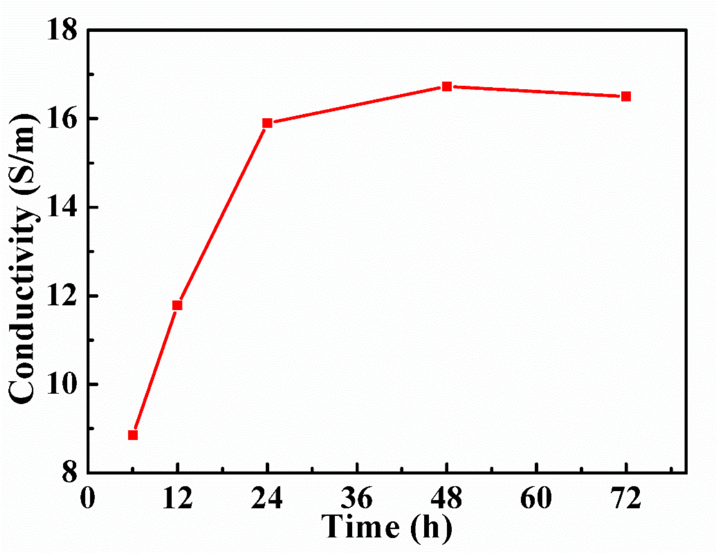

Figure S9. Conductivity curves of different deposition time for PAN nanofiber core-spun yarns.

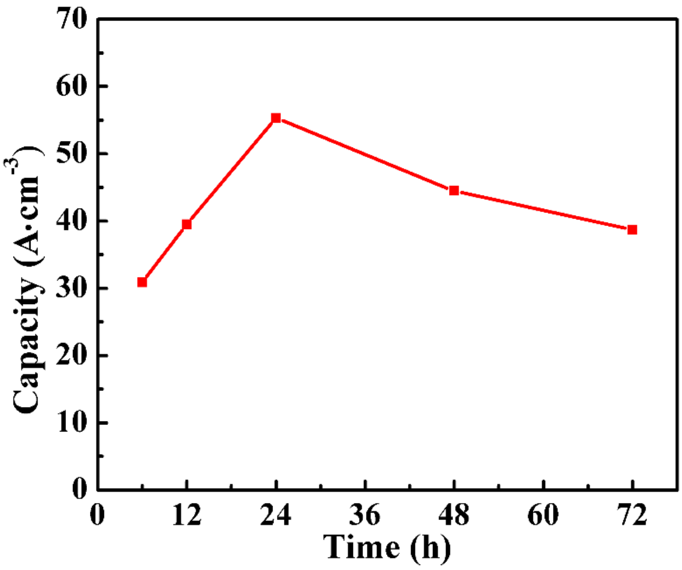

Figure S10. Volumetric capacity curve after in-situ deposition of PEDOT: PSS for different hours.

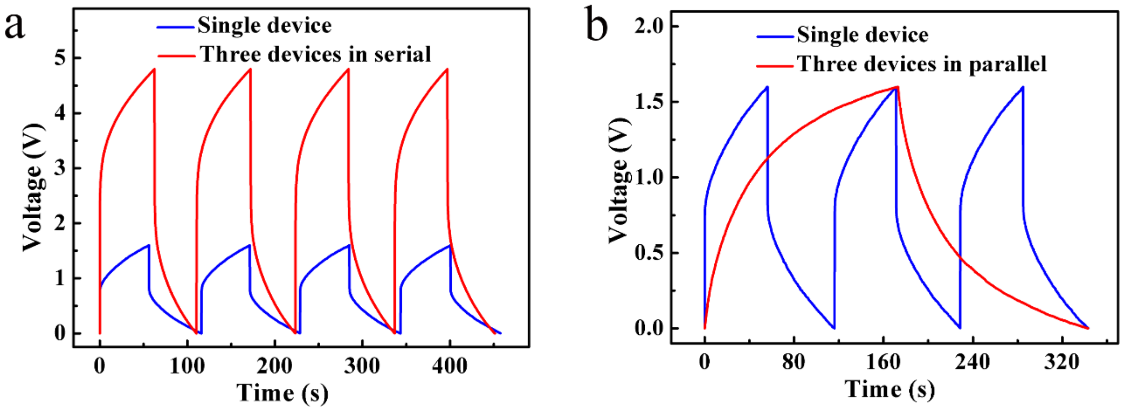

Figure S11. Galvanostatic charge and discharge curves of three SC yarns in series (**a**) and parallel (**b**).

Supplementary Tables

Table S1. Nitrogen absorption and desorption data for different stage's materials.

	Surface area($m^2 \cdot g^{-1}$)	Pore volume($cm^3 g^{-1}$)	Pore size(nm)
Ni-coated yarns	1.755	0.002	1~50
PAN/Ni-coated yarns	3.549	0.012	1~50
PNF/NiC-6	4.543	0.003	1~50
PNF/NiC-12	5.180	0.004	1~50
PNF/NiC-24	5.329	0.018	1~50
PNF/NiC-48	2.089	0.001	1~50
PNF/NiC-72	0.893	0.001	1~50

Table S2. Specific capacitance of yarn electrode material deposited PEDOT: PSS for different hours.

Deposition time(h)	6	12	24	48	72
Volumetric capacitance ($F \cdot cm^{-3}$)	30.88	39.52	55.36	44.48	38.72

Table S3. Migration resistance of capacitors for different deposition time of PEDOT: PSS.

Deposition time(h)	6	12	24	48	72
Resistance(Ω)	76	43	16	47	64

© 2019 by the authors. Submitted for possible open access publication under the terms and conditions of the Creative Commons Attribution (CC BY) license (http://creativecommons.org/licenses/by/4.0/).
